# Prescribing patterns of asthma controller therapy for children in UK primary care: a cross-sectional observational study

**DOI:** 10.1186/1471-2466-10-29

**Published:** 2010-05-14

**Authors:** Mike Thomas, Tarita Murray-Thomas, Tao Fan, Tim Williams, Stephanie Taylor

**Affiliations:** 1 Department of General Practice and Primary Care, University of Aberdeen, Foresterhill Health Centre, Westburn Road, Aberdeen, UK; 2 General Practice Research Database, London, UK; 3 Outcomes Research, Merck & Co, Inc., Whitehouse Station, NJ, USA

## Abstract

**Background:**

Asthma management guidelines recommend a stepwise approach to instituting and adjusting anti-inflammatory controller therapy for children with asthma. The objective of this retrospective observational study was to describe prescribing patterns of asthma controller therapies for children in a primary care setting.

**Methods:**

Data from the UK General Practice Research Database were examined for children with recorded asthma or recurrent wheezing who, from September 2006 through February 2007, were ≤ 14 years old at the time of a first asthma controller prescription after ≥ 6 months without a controller prescription. We evaluated demographic characteristics, asthma duration, comorbidities, asthma-related health care resource use, and prescribed daily dose of controller medication. In addition, physicians for 635 randomly selected patients completed a survey retrospectively classifying asthma severity at the prescription date and describing therapy and health care utilization for 6 prior months.

**Results:**

We identified 10,004 children, 5942 (59.4%) of them boys, of mean (SD) age of 8.0 (3.8) years. Asthma controller prescriptions were for inhaled corticosteroid (ICS) monotherapy for 9059 (90.6%) children; ICS plus long-acting β2-agonist (LABA) for 698 (7.0%); leukotriene antagonist monotherapy for 91 (0.9%); ICS plus leukotriene antagonist for 55 (0.6%); and other therapy for 101 (1.0%), including 45 (0.45%) children who were prescribed LABA as monotherapy. High doses of ICS (> 400 μg) were prescribed for 44/2140 (2.1%) children < 5 years old and for 420/7452 (5.6%) children ≥ 5 years. Physicians reported asthma severity as intermittent for 346/635 (55%) patients and as mild, moderate, and severe persistent for 159 (25%), 71 (11%), and 11 (2%), respectively (severity data missing for 48 [8%]). The baseline characteristics and controller therapy prescriptions of the survey cohort were similar to those of the full cohort.

**Conclusions:**

Physician classifications of asthma severity did not always correspond to guideline recommendations, as leukotriene receptor antagonists were rarely used and high-dose ICS or add-on LABA was prescribed even in intermittent and mild disease. In UK primary care, monotherapy with ICS is the most common controller therapy at all levels of asthma severity.

## Background

Asthma usually begins in the first years of life and is the most common chronic disease of childhood in developed countries [1,2]. In the United Kingdom (UK) and other developed countries there are some indications that the incidence of childhood asthma may have peaked at the end of the last century [2-4]. Nonetheless, a large proportion of children are affected-an estimated 1 in 11 children in the UK [5]-and asthma is a common condition usually managed by general practitioners (GPs).

Anti-inflammatory therapy is the cornerstone of pharmacotherapy for persistent asthma to counter the chronic airway inflammation that characterises this condition. International and British asthma management guidelines recommend a stepwise approach to instituting and adjusting daily controller (preventer) anti-inflammatory therapy, beginning with the dose of inhaled corticosteroid (ICS) appropriate to the severity of asthma [6-9]. For children 5 years and older with worsening symptoms, or as necessary to achieve asthma control, options for controller therapy include increasing the ICS dose or add-on therapy with inhaled long-acting β2-agonist (LABA) or leukotriene receptor antagonist (LTRA). For children under 5, the British guidelines recommend an LTRA for those who cannot use ICS and as the sole recommended add-on therapy to ICS [6,7], and a recent consensus report places greater emphasis on using LTRA as an alternative to ICS at all ages [1]. Short-acting bronchodilators are recommended for relief of mild intermittent asthma and should always be available to treat asthma symptoms as needed for children with persistent asthma who are using regular controller therapy [6-9].

The objective of this cross-sectional study was to describe prescribing patterns of asthma controller therapies for children in the UK primary care setting who had not received a controller therapy prescription in the prior 6 months and to explore the clinical and demographic characteristics influencing the choice of medication prescribed, as well as health care utilisation 6 months prior. In addition, we describe these parameters for a sub-cohort of children whose asthma severity at the time of initiating controller therapy was assessed retrospectively. Table 1 summarises the treatment recommendations for childhood asthma that were current at the time of this study [7,9]. These recommendations have remained little changed since then [6,8].

**Table 1:** Summary of treatment recommendations for childhood asthma according to 2005 Global Initiative for Asthma (GINA) guidelines [9] and 2005 British Thoracic Society (BTS) guidelines [7]

2005 GINA Guidelines for adults & children > 5 years old*		2005 BTS Guidelines for children 5-12 years old*	
GINA Step 1 Intermittent asthma	No controller medication necessary Short acting β2 agonist as needed	BTS Step 1 Mild intermittent asthma	Short acting β2 agonist as required
GINA Step 2 Mild persistent asthma	Low-dose ICS (*or *SR-theophylline; cromone; or leukotriene modifier)	BTS Step 2 Regular preventer therapy	ICS 200-400 μg/d†: 200 μg/d is a reasonable starting dose [for those < 5 y, use LTRA if cannot use ICS]
GINA Step 3 Moderate persistent asthma	Low- to medium-dose ICS *plus *inhaled LABA (*or *medium-dose ICS plus SR-theophylline, plus oral LABA, or plus leukotriene modifier; OR high-dose ICS) [for those ≤ 5 y, medium-dose ICS]	BTS Step 3 Add-on therapy	Add inhaled LABA; if poor response try ICS 400 μg/d; if poor response, add LTRA or SR-theophylline (ICS dose up to 400 μg/d) [for those 2-5 y, trial of LTRA]
GINA Step 4 Severe persistent asthma	High-dose ICS *plus *LABA *plus *1 or more of the following if needed: SR-theophylline, leukotriene modifier, oral LABA, oral steroid, anti-IgE [for those ≤ 5 y, high-dose ICS *plus *add-on Rx if needed]	BTS Step 4 Persistent poor control	Increase ICS dose to 800 μg/d Consider adding the following if needed: LTRA, theophylline, SR-β2 agonist tablets [for those < 5 y, refer to respiratory paediatrician]
--	--	BTS Step 5 Continuous or frequent use of oral steroids	Use daily steroid tablet in lowest dose; maintain high-dose ICS; refer to respiratory paediatrician

ICS = inhaled corticosteroid; LABA = long-acting β2-agonist; LTRA = leukotriene receptor antagonist; SR = slow-release

*Guidelines are the same for children ≤ 5 years (GINA) or < 5 years old (SIGN/BTS) unless specified.

† ICS doses are the beclometasone dipropionate equivalent doses.

## Methods

### Data source

Data regarding diagnosis, prescriptions, hospitalisations, co-morbidities, and demographic and clinical characteristics were derived from the General Practice Research Database (GPRD). The GPRD [10] is a large computerised database containing anonymised longitudinal medical records from approximately 500 primary care practices throughout the UK (England, Scotland, Northern Ireland, and Wales). Well-accepted and validated for respiratory epidemiologic research in primary care [11,12], the GPRD contains records for over 13 million patients; active records are available for 3.6 million patients, equivalent to ~5.5% of the population of the UK.

Approval was given for the use of GPRD data for this study by the UK Medicines and Healthcare products Regulatory Agency (MHRA) Independent Scientific Advisory Committee.

### Patients

We identified children who, from September 2006 through February 2007, were ≤ 14 years old at the time of a recorded prescription for asthma controller therapy (defined below) and who had no record of asthma controller medication during the 6 months before date of the prescription (index date). As further evidence of asthma recorded in the database, children had to have either a prior medical diagnosis of asthma; ≤ 2 episodes of wheezing at least 28 days apart; or ≥ 2 prescriptions for a short-acting β2 agonist (SABA) or ipratropium bromide at least 28 days apart. We excluded children with a history of bronchopulmonary dysplasia, cystic fibrosis, or any chronic pulmonary disorder other than asthma or wheezing.

In addition, we randomly selected 792 children from the full study cohort for inclusion in a physician-based cross-sectional structured survey to assess asthma severity in relation to controller therapy. (A total of 900 children were originally selected, and 108 of these were ultimately excluded as these children had been prescribed oral corticosteroids, not considered a controller therapy.) Physicians were asked to complete the survey questionnaire (Additional file 1), retrospectively classifying asthma severity at the index date and 6 months prior on the basis of clinical data held electronically and otherwise. Classification of asthma severity as per the Global Initiative for Asthma (GINA) 2005 guidelines [9] was provided to GPs to assist with the rating of severity. Completed surveys were returned to the GPRD and entered into a dedicated database. Physicians were compensated for completing the survey questionnaire.

### Data extraction and analysis

We captured the following asthma controller therapies in the database: ICS, inhaled LABA, ICS and LABA combinations, sustained release β2-agonists, sustained release theophylline, cromones, and antileukotrienes. We also tabulated prescriptions for SABA on the index date, as well as for 6 months before and 12 months after. Database criteria for defining an asthma diagnosis and wheezing are listed in Additional file 2.

We tabulated demographic characteristics, smoking history, asthma duration, and the prescribed daily dose of controller medication at the index date for each patient, stratified by controller therapy type and, for the survey cohort, asthma severity. In addition, we collected data on respiratory and allergic comorbidities and on asthma-related health care resource use for up to 24 months before the index date.

### Statistical methods

Descriptive statistics were used to characterise the distribution of demographic factors, controller medication prescribing at baseline, SABA prescriptions, comorbidities, and resource use. For categorical variables, the proportion of patients with the variable(s) of interest was calculated and compared using χ^2 ^analysis or Fisher's exact test. For quantitative variables, comparisons were made using Student's *t *test, Wilcoxon rank-sum test, and analysis of variance (ANOVA) where required. All statistical analysis was conducted using STATA version 10 software (StataCorp LP, College Station, TX, USA).

## Results

### Full cohort and survey cohort-demographic and clinical characteristics at baseline (index date of controller therapy prescribing)

We identified 10,004 children 0-14 years old in the GPRD who were prescribed asthma controller therapy between September 1, 2006, and February 28, 2007, and who had no controller prescription during the prior 6 months. Their mean age was 8 years; 806 (8%) patients were 2 years or younger and the other patients were evenly distributed over the age range of 3-14 years; overall, 30% were 5 years or younger; 59% were boys (Table 2). The duration of asthma, as recorded in the GPRD, was ≤ 3 years for slightly over half of patients; 4-7 years for 28%, and ≥ 8 years for 19%. Approximately half of children were identified as having asthma because of a physician's diagnosis in the database; approximately 40% were identified on the basis of SABA or ipratropium prescriptions; and < 10% were identified because of a record of recurrent wheezing.

**Table 2:** Characteristics of children new to asthma controller therapy, stratified by full cohort and survey responder cohort

Characteristic	Full cohort (n = 10,004)	Survey responders (n = 635)	p value
Mean age (SD), yr	8.0 (3.8)	8.1 (3.8)	0.35
Age 0-2 yr, n (%)	806 (8.1%)	51 (8.0%)	
Age 3-5 yr, n (%)	2155 (21.5%)	143 (22.5%)	
Age 6-8 yr, n (%)	2307 (23.1%)	126 (19.8%)	
Age 9-11 yr, n (%)	2402 (24.0%)	165 (26.0%)	
Age 12-14 yr, n (%)	2334 (23.3%)	150 (23.6%)	
Male sex, n (%)	5942 (59.4%)	365 (57.5%)	0.29
Smoking status			0.22
Non smoker	5651 (56.5%)	366 (57.6%)	
Smoker	532 (5.3%)	31 (4.9%)	
Ex smoker	77 (0.8%)	6 (0.9%)	
Passive smoker	152 (1.5%)	3 (0.5%)	
Unknown smoking status	3592 (35.9%)	229 (36.1%)	
BMI, n	5816	393	
Mean BMI (SD)	17.3 (5.3)	17.1 (5.0)	0.32
Median BMI (range)	16.6 (5.0-49.6)	16.7 (5.2-47.7)	0.75
Asthma duration: mean (SD), yr	4.3 (3.6)	4.4 (3.6)	0.56
Median (range), yr	3.7 (0.0-15.0)	3.9 (0.0-14.0)	0.62
Asthma controller therapy prescription at index date, n (%)			0.437
ICS monotherapy	9059 (90.6%)	575 (90.6%)	
LABA in fixed dose combination	574 (5.7%)	43 (6.8%)	
ICS + LABA	124 (1.2%)	4 (0.6%)	
LTRA monotherapy	91 (0.9%)	6 (0.9%)	
ICS + LTRA	55 (0.6%)	1 (0.2%)	
Other	101 (1.0%)	6 (0.9%)	

BMI = body mass index; ICS = inhaled corticosteroids; LABA = long-acting β2-agonists (in fixed dose combination is with ICS); LTRA = leukotriene receptor antagonists.

During the 6 months before the index date, 396 (4%) children were prescribed an oral corticosteroid, and 525 (5%) received antibiotics for a lower respiratory tract infection. Lung function test results, usually as peak expiratory flow rate, were recorded during the 6 months preceding the index date for 974 (10%) children. On the index date, lung function testing was performed for 1657 (17%) children, recorded as peak expiratory flow rate for 1581 (16%) children.

Of the 792 surveys sent to GPs, 635 (80%) were completed. The baseline characteristics of the surveyed responder cohort were similar to those of the full cohort (see Table 2) as well as to those of the survey nonresponders (data not shown). Physicians completed the asthma severity classification for all but 48 of 635 (8%) patients: just over half (346/635, 55%) of patients were reported as having intermittent asthma, and 159 (25%), 71 (11%), and 11 (2%) were reported as having mild, moderate, and severe persistent asthma, respectively.

### Full cohort and survey cohort-index therapy prescription patterns and doses of ICS monotherapy

Most children (91%) were prescribed ICS monotherapy as asthma controller therapy (Table 2). Of those children with defined dosing information, high doses of ICS (> 400 but ≤ 800 μg/day of chlorofluorocarbon (CFC)-beclometasone or equivalent) were prescribed for 33/2140 (1.5%) children < 5 years old and for 240/7452 (3.2%) children ≥5 years. The highest doses of ICS (> 800 μg/day of CFC-beclometasone or equivalent) were prescribed for 11/2140 (0.5%) < 5 years old and 180/7452 (2.4%) ≥ 5 years old. (Doses of ICS were standardised to the CFC-beclometasone dipropionate [CFC-BDP] equivalent dose using the following ratios relative to CFC-BDP: budesonide [BUD], fluticasone propionate [FP], BDP in solution [QVAR^®^, Teva UK], and mometasone [MOM] at BDP:BUD:FP:QVAR:MOM = 1:1:2:2:2, as per British asthma guidelines [6,7].) Included among the "other" therapy group, 45 (0.45%) children were prescribed LABA as monotherapy.

**Figure 1:**
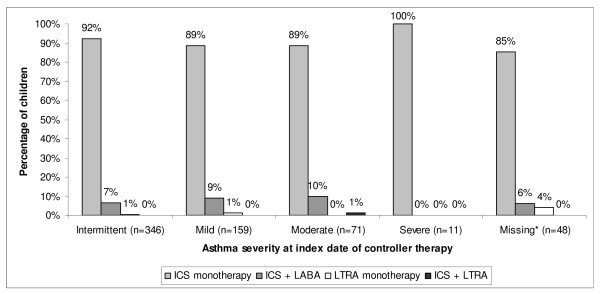
**Asthma controller therapy prescription, by asthma severity, for 635 children ≤ 14 years old**. Children had not been prescribed an asthma controller medication in the prior 6 months. Data were derived from the UK General Practice Research Database. Asthma severity was defined by physicians according to GINA guidelines 2005 [9]. *Missing asthma severity classification. ICS = inhaled corticosteroid; LABA = long-acting β2-agonist; LTRA = leukotriene receptor antagonist

For the surveyed responder cohort (those with known asthma severity), monotherapy with ICS was prescribed for all of the patients with severe asthma and approximately 90% of patients in the other severity classifications (Figure 1). ICS and LABA either in fixed dose combination or in separate inhalers were prescribed to 8%, 9%, and 10% of children with intermittent, mild, and moderate asthma, respectively. Only five of the children with reported asthma severity received an LTRA, administered as monotherapy for four. Controller therapy prescriptions and doses are summarised in Table 3.

**Table 3:** Prescribed daily dose of controller medication at cohort entry by asthma severity--prescriptions with defined dosage information for survey cohort

	Asthma severity classification				
	
	Intermittent (n = 346)	Mild (n = 159)	Moderate (n = 71)	Severe (n = 11)	Missing (n = 48)
Total no. prescriptions	351	164	75	13	51
Missing	7 (2.0%)	5 (3.0%)	1(1.3%)	0	3 (5.9%)
Beclometasone, no.	267	123	59	10	35
Mean dose (SD)	261 (126)	230 (112)	258 (107)	230 (95)	269 (149)
Budesonide, no.	33	11	4	2	3
Mean dose (SD)	218 (101)	364 (229)	350 (100)	300 (141)	400 (346)
Fluticasone, no.	19	8	3	1	1
Mean dose (SD)	216 (75)	150 (54)	167 (58)	100	200
Montelukast, no.	2	3	1	0	4
Mean dose (SD)	5 (0)	5 (1)	4	--	5 (1)
Salmeterol, no.	4	1	1	0	1
Mean dose (SD)	88 (25)	50	100	--	100
FDC Budesonide	1	3	1	0	3
Mean dose (SD)	1600	267 (116)	400	--	333 (116)
FDC-Formoterol	1	3	1	0	3
Mean dose (SD)	48	16 (7)	24	--	20 (7)
FDC-Fluticasone	3	10	5	0	16
Mean dose (SD)	400 (173)	180 (42)	240 (152)	--	294 (232)
FDC-Salmeterol	3	10	5	0	16
Mean dose (SD)	100 (0)	90 (21)	90 (22)	--	94 (25)

FDC = fixed-dose combination

Doses are actual doses as prescribed (μg/d).

Prescriptions for SABA were written for 31% of all patients during the 6 months before their index controller prescription; for 75% on the index date; and for 72% during the 12 months after the index date. Similar overall proportions of patients in the survey cohort as in the full study cohort received SABA prescriptions, as shown in Table 4. The proportions of patients receiving SABA prescriptions varied significantly (P < 0.01) among controller therapy groups, although no obvious pattern was evident, as well as among asthma severity groups excepting on the index date (Table 4).

**Table 4:** Short-acting β-agonist prescriptions

	6 months prior	On index date	12 months after
No. prescriptions, median (range)	1 (1-13)	1 (1-3)	2 (1-26)
Patients with prescriptions, by controller therapy, n (%)			
ICS monotherapy (n = 9059)	2805 (31.0)*	6876 (75.9)*	6515 (71.9)*
ICS + LABA (n = 698)	174 (24.9)	497 (71.2)	519 (74.4)
ICS + LTRA (n = 55)	13 (23.6)	44 (80.0)	49 (89.1)
LTRA monotherapy (n = 91)	38 (41.8)	30 (33.0)	69 (75.8)
Other (n = 101)	31 (30.7)	62 (61.4)	82 (81.2)
Total (n = 10,004)	3061 (30.6)	7509 (75.1)	7234 (72.3)
Survey cohort: patients with prescriptions, by asthma severity, n (%)			
Intermittent (n = 346)	87 (25.1)*	274 (79.2)	241 (69.7)*
Mild persistent (n = 159)	57 (35.9)	118 (74.2)	129 (81.1)
Moderate persistent (n = 71)	30 (42.3)	52 (73.2)	62 (87.3)
Severe persistent (n = 11)	6 (54.6)	9 (81.8)	9 (81.8)
Missing classification (n = 48)	13 (27.1)	31 (64.6)	36 (75.0)
Total (n = 635)	193 (30.4)	484 (76.2)	477 (75.1)

*P < 0.01 for comparisons among controller therapy groups or asthma severity groups.

### Full cohort-characteristics according to asthma controller therapy

Additional file 3 summarises baseline characteristics and prior health care resource utilisation stratified according to asthma controller therapy. We found significant differences among controller therapy groups for mean age, age distribution, and asthma duration. Children who received ICS and LABA either in fixed dose combination or in separate inhalers tended to be older and to have a longer duration of asthma than those in other treatment cohorts (see Additional file 3). The prevalence of comorbidities and health care resource utilisation before the index date also differed significantly among controller therapy groups.

## Discussion

We found that the most common asthma controller therapy recorded from September 2006 through February 2007 in the UK GPRD for children aged 14 years and younger with no controller prescription during the prior 6 months was monotherapy with ICS, prescribed for over 90% of children. This is in line with UK guidelines [6,7]. However, the prescribing pattern for controller therapy in the survey cohort did not vary much across asthma severity categories as classified by physicians. Monotherapy with ICS, and beclometasone in particular, was the most common prescription for children in each severity classification; mean daily doses of ICS did not change substantially with asthma severity. Moreover, combination therapy with ICS and LABA was prescribed in similar proportions for patients with intermittent asthma (7.7%) as for those with moderate asthma (9.9%).

Results of this observational study indicate that prescribing practices did not always correspond to recommendations of asthma management guidelines. Both international and British asthma guidelines recommend stepwise introduction of controller therapy, typically beginning with low-dose ICS, defined in British guidelines as 200 μg/day of CFC-beclometasone dipropionate or equivalent for children 12 years and younger and 400 μg/day for older children [6,7]. We found, instead, that substantial numbers of children had no prescription for ICS monotherapy during the prior 6 months and were prescribed combination therapy with ICS and LABA or doses of ICS as high as 800 μg/day. Of note, doses of > 800 μg/day are outside guideline and licensing parameters, as are doses of > 400 μg/day for the younger age group [7]. The high doses of ICS, recommended for use only after careful thought in children with severe persistent disease [7], were sometimes prescribed to children classified as having intermittent or mild persistent asthma.

The pattern of prescribing of combination therapy with ICS and LABA, which asthma guidelines recommend for patients with moderate or severe persistent asthma, was further evidence that recalled asthma severity and therapy were not consistent. Combination ICS-LABA therapy was prescribed for 7% of patients, most of whom were classified as having intermittent or mild persistent asthma; these children tended to be older and to have a longer duration of asthma. Similarly, in a recent study of insurance claims in the US [13], over half of children (55%) receiving a first prescription for fluticasone propionate and salmeterol in fixed-dose combination had no record of pharmacy or medical claims during the prior year that would appear to warrant ICS-LABA controller therapy. In that study, as in the present study, a small percentage of children were prescribed LABA monotherapy, which all guidelines strongly recommend against and which lies outside the licence for this therapy class [6,8].

Recent studies have suggested that LABA use is increasing in children, particularly as a first-line combination inhaler [14,15]. Although this may be appropriate for some children [16,17], many could be managed with ICS alone, and recent concerns have been expressed by the US regulatory agency as to the increasing use of LABAs in this age group, with the FDA tightening the rules governing the use of this class of agents [18] and leading experts calling for caution in their use [19].

Over 50% of patients in the survey cohort were classified as having intermittent asthma and thus, assuming the classifications were correct, may not have asthma of sufficient severity to warrant controller therapy. Both the 2005 GINA guidelines and the British asthma management guidelines in effect at the time [7,9], as well as current guidelines [6,8], recommend as-needed short-acting bronchodilators as reliever therapy for intermittent asthma; regular controller therapy is not initiated until asthma symptoms become more regular or troublesome. Guidelines recommend that patients on controller therapy also have reliever bronchodilators available. We found that approximately one third of patients had prescriptions for SABA before the index date, and three quarters received SABA prescriptions on the index date.

Of the children with severe asthma, none were prescribed additional therapy as add-on to ICS, although almost half received oral corticosteroids on the index date; these patients were too few in number (n = 11) to enable us to draw conclusions from the findings. Leukotriene antagonists were not commonly prescribed. Finally, objective measures of airway obstruction (spirometry or peak flow measurement) were recorded for fewer than one fifth of children.

Our findings point to sub-optimal clinical practice but are not unusual. Clinical guidelines, including asthma management guidelines, are often not followed in general practice [20-23]. Other authors have found evidence of off-label prescribing and high-dose ICS prescribing for childhood asthma in the UK [14,24-27]. Many reasons have been proposed for the failure to follow guidelines, including incomplete dissemination, lack of agreement, time pressures, inertia of prior practice, and, for the youngest patients, lack of appropriate formulations [28]. In a recent Danish study, specialists were more likely than GPs to provide guideline-adherent care to children with asthma; nonetheless, overall, only one quarter of children in the study received care in accordance with the guidelines [29].

Guidelines are formulated to present busy clinicians with a summary of up-to-date evidence of the effectiveness of different treatment options, and so to aid therapeutic decisions. The British asthma guidelines are distributed to all clinicians treating asthma in the UK, where this study occurred, and are accepted as the standard for asthma treatment. These guidelines recommend that "...departures from the national guideline....should be fully documented in the patient's case notes..." and justified [6,7]. Although it is recognized that following guidelines does not automatically lead to optimal outcomes, studies show that outcomes can indeed be improved by implementing recommendations from clinical practice audits in line with guideline recommendations [30,31]. Moreover, guideline recommended management of acute asthma is associated with improved outcomes [32].

Our findings describe prescribing practices specific to the UK. Turner and coworkers [14] analysed longitudinal trends in asthma therapies recorded in the GPRD from 1992-2004 for children 0-11 years old with an asthma diagnosis: in 2004, approximately 9 of 10 children received monotherapy with ICS and 1 in 10, add-on therapy to ICS, most commonly LABA. Prior observational studies, while different in design to the present study and thus not directly comparable, depict quite different prescribing patterns for childhood asthma controller therapy in other countries. Relative to our findings for the UK, in the US (1999-2000) montelukast was prescribed more frequently [33]; in Taiwan (2002) ICS were prescribed less frequently [34]; and in Norway (2004) combination ICS plus LABA was prescribed more frequently [35].

The GPRD is a large, well-validated database that records real-life prescribing practices and is relevant and accepted for studying childhood asthma in the UK, as most of these children are managed in primary care [11,36,37]. Our findings, as for all database studies, are dependent on the accuracy and completeness of recording in the database [38]. Another study limitation is the possibility that our database definitions incorrectly categorised children with regard to asthma diagnosis. Moreover, asthma in children under the age of 5 or 6 years is difficult to diagnose and is essentially a provisional diagnosis, as wheezing often resolves with age [1,39]. Approximately 30% of children in this study were aged 5 and younger. Despite the difficulty in diagnosing asthma in young children, we believe that observational research of how wheezing children are treated in real-world practice is important, as international and national guidelines advise GPs on the ways to diagnose and manage asthma in younger children, and GPs make the diagnosis on a frequent basis. Wheezing illnesses are common in this age group, and previous research has suggested that management of these younger children in whom a clinical diagnosis of asthma has been made does not always correspond to guideline recommendations [14,26].

An important study limitation is the possibility of missing data; for the survey sub-study we were unable to link data on 51 prescriptions to asthma severity. Moreover, it cannot be assumed that drugs as prescribed in the database were also dispensed or consumed. In addition, our findings could reflect seasonal trends, as we gathered data for 6 months and not a full year. When available, we gathered information on comorbid conditions for the prior 24 months; however, some children did not have data going back 24 months, e.g., those under 12 months old, thus the patient percentages for these data may not be comprehensive. Finally, asthma severity, based on 2005 GINA guidelines [9], was classified retrospectively by physicians completing the survey; therefore, this tool to guide clinicians in determining asthma severity was not available to them at the time of prescribing and, moreover, the results could have been biased by incomplete recall.

## Conclusions

This cross-sectional study has enabled us to describe UK primary care prescribing practices in late 2006 and early 2007 for asthma controller therapy for children with recorded asthma or recurrent wheezing and no controller therapy in the prior 6 months. We found that physician classifications of asthma severity did not always correspond to guideline recommendations for prescribing controller therapy. Children classified as having intermittent asthma were prescribed controller therapy with ICS, sometimes at high doses, and those with intermittent and mild asthma received combination therapy with ICS and LABA. High doses of ICS were prescribed without a prior low-dose ICS prescription for some children, and combination therapy with ICS and LTRA was rarely prescribed. In UK primary care, despite endorsement in local guidelines, children with asthma are seldom treated with LTRA, and monotherapy with ICS is the most common controller therapy at all levels of severity.

## Competing interests

Neither Mike Thomas nor any member of his close family has any shares in pharmaceutical companies. In the last 3 years he has received fees for acting as a consultant for MSD, Schering, and GSK and has received speaker's honoraria for speaking at sponsored meetings from the following companies marketing respiratory and allergy products: Astra Zeneca, Boehringer Ingleheim, GSK, MSD, Schering-Plough, Teva. He has received honoraria for attending advisory panels with Altana, Astra Zeneca, BI, GSK, MSD, Merck Respiratory, Schering-Plough, Teva. He has received sponsorship to attend international scientific meetings from GSK, MSD, Astra Zeneca. He has received funding for research projects from GSK, MSD, Astra Zeneca. He holds a research fellowship from Asthma UK.

Tao Fan and Stephanie Taylor are employees of Merck & Co., Inc.

Tarita Murray-Thomas and Tim Williams are GPRD members of staff and have no competing interests to declare.

## Authors' contributions

MT participated in the study design, data analysis, results interpretations, and manuscript development. T M-T participated in the study design, data analysis, results interpretations, and manuscript development. TF participated in the study design, data analysis, results interpretations, and manuscript development. TW participated in the study design, data analysis, results interpretations, and manuscript development.

ST participated in the study design, data analysis, results interpretations, and manuscript development. All authors read and approved the final manuscript.

## Pre-publication history

The pre-publication history for this paper can be accessed here:

http://www.biomedcentral.com/1471-2466/10/29/prepub

## Supplementary Material

Additional file 1 **RE-CLIC Survey Physician Questionnaire**. physician survey form used in the study. Click here for file

Additional file 2 **Criteria for identifying a medical diagnosis of asthma or wheezing**. Criteria for identifying a medical diagnosis of asthma or wheezing criteria for identifying children with asthma or wheezing in the GPRD. Click here for file

Additional file 3 **Table S5 for the manuscript Table S5: Characteristics of 10,004 children new to asthma controller therapy, stratified by treatment group (column percentages)**. original table S5. Click here for file
