# Supplementary material for: Prescribing patterns of asthma controller therapy for children in UK primary care: a cross-sectional observational study
Source: BMC Pulm Med. 2010 May 14;10:29. doi: 10.1186/1471-2466-10-29 (PMC2882363; doi:10.1186/1471-2466-10-29)
Supplement: Additional file 1 — RE-CLIC Survey Physician Questionnaire. physician survey form used in the study. [file 1471-2466-10-29-S1.DOC]

RE-CLIC Survey Physician Questionnaire

**Diagnostic Criteria**

| 1. Please check (✓) one box for each of the following | | |
| --- | --- | --- |
| 1. Patient was less than 15 years old on the **index date*** of **‘date field’**. | Yes ❑ | No ❑ |
| 1. Patient had **at least one** of the following present during the study period -1^st^ September 2006 - 28^th^ February 2007 inclusive    - a diagnosis of asthma    - 1 episode of hospitalisation, out-patients visit or A&E visits related to   asthma   - - 2 episodes of wheezing at least 28 days apart   - 2 episodes of hospitalisation, out-patients visit or A&E visits related to wheezing at least 28 days apart   - 2 Short acting beta-agonist^†^ or ipatropium bromide prescriptions at least   28 days apart  **^†^ Short acting beta-agonist : salbutamol, fenoterol, bambuterol, terbutaline or**  **pirobuterol** | Yes ❑ | No ❑ |
| 1. Patient in need of daily asthma preventer therapy.   Please refer to **Appendix I** for the list of asthma preventer therapies of  interest | Yes ❑ | No ❑ |
| 1. Patient was prescribed an asthma preventer medication on ‘**date field’** | Yes ❑ | No ❑ |
| 1. Was this patient registered with the surgery for at least 12 months prior to study period (01 September 2006 and 28 February 2007). | Yes ❑ | No ❑ |
| 1. The surgery is the primary source of asthma prevention therapy for the patient. | Yes ❑ | No ❑ |
| 1. Patient has any chronic pulmonary disorders other than asthma or wheezing (e.g. cystic fibrosis). | Yes ❑ | No ❑ |

*Index date is the earliest date during 01 Sept 2006 -28 Feb 2007 inclusive when an asthma ***preventer*** medication was prescribed

**Patient Demographics and Co-morbidities**

| 2. Please check (✓) the appropriate box | |
| --- | --- |
| Month and year of birth | ______/_____ (mm/yyyy) |
| Gender | ❑ male ❑ female |
| Has patient ever smoked  If yes, is the patient currently smoking? | ❑ yes ❑ no  ❑ yes ❑ no |
| Does either of the patient’s parents/guardians currently smoke | ❑ yes ❑ no |

| 3. Has patient had any of the following background or concomitant diseases?  Please check (✓) appropriate boxes | | | | |
| --- | --- | --- | --- | --- |
| Time Period | On **‘date field’** | | within 2 years prior to **‘date field’** | |
| Condition | Yes | No | Yes | No |
| ❑ Allergic rhinitis/hay fever*  Check one circle that best describes the type of allergic rhinitis in the patient  🔾 Seasonal 🔾 Perennial with seasonal exacerbations 🔾 Perennial |  |  |  |  |
| ❑ Atopic dermatitis |  |  |  |  |
| ❑ Sinusitis |  |  |  |  |
| ❑ Otitis media |  |  |  |  |
| ❑ Gastro-oesophageal reflux |  |  |  |  |

*Symptoms of allergic rhinitis are: rhinorrhoea, nasal obstruction, nasal itching, sneezing; **any** required.

## ASSESSMENT OF ASTHMA SEVERITY USING *ADAPTED* GINA GUIDELINES 2005

**Please use Appendix II to assess asthma severity according to *adapted* GINA guidelines.**

| 1. | Indicate the date when asthma was first diagnosed in this patient: ________________ | | | |
| --- | --- | --- | --- | --- |
|  | **PLEASE USE APPENDIX II FOR THE ASSESSMENT OF ASTHMA SEVERITY** | | | |
| 2. | Severity on the ‘date field’ | | **Severity 6 mths prior to ‘date field’** | |
|  | ❑ | Intermittent | ❑ | Intermittent |
|  | ❑ | Mild persistent | ❑ | Mild persistent |
|  | ❑ | Moderate persistent | ❑ | Moderate persistent |
|  | ❑ | Severe persistent | ❑ | Severe persistent |
| 3. | Indicate the most recent FEV_1_ available (and date of recording) in the record for this patient: between Sept 06 and Feb 07______________ (value) _______________(date in mm/yyyy) | | | |
| 4. | Indicate the most recent PEF available (and date of recording) in the record for this patient: between Sept 06 and Feb 07______________ (value) _______________(date in mm/yyyy) | | | |

## ASTHMA PREVENTER TREATMENT INITIATION

| Indicate below **all** medicines that you prescribed for the patient for **asthma** on **‘date field’**. | | | | |
| --- | --- | --- | --- | --- |
| Drug proprietary name  (Brand name and presentation) | **Route** | **Number of days** | **Total Daily Dosage** | |
|  |  |  | **Dose per unit** | **Number of units** |
|  |  |  |  |  |
|  |  |  |  |  |
|  |  |  |  |  |
|  |  |  |  |  |
|  |  |  |  |  |
|  |  |  |  |  |

Please turn over if required

**APPENDIX I: LIST OF ASTHMA PREVENTER THERAPIES**

| **Systemic Glucocorticosteroid *(Oral or Parenteral)***  Hydrocortisone  Methylprednisolone  Prednisolone  Prednisone |
| --- |
| **Inhaled Glucocorticosteroid**  Beclometasone dipropionate  Budesonide  Fluticasone  Mometasone Furoate  Ciclesonide  Flunisolide  Triamcinolone Acetonide |
| **Sustained Release β_2_-Agonist**  Salbutamol  Terbutaline Sulphate  **Long acting β_2_-Agonist (Inhaled)**  Formoterol Fumarate  Salmeterol |
| **Long Acting β_2_-agonist/ ICS combinations**  Budesonide & Formoterol fumarate  Fluticasone & Salmeterol |
| **Sustained Release Theophylline**  Theophylline  Aminophylline |
| **Cromones**  Sodium Cromoglicate  Nedocromil Sodium |
| **Antileukotrienes**  Montelukast  Zafirlukast |

**Table 1: ASTHMA SEVERITY ASSESSMENT BASED ON**

**CLINICAL FEATURES – GINA 2005** ^†^

| **Step 1: Intermittent**  Symptoms less than once a week  Brief exacerbations  Nocturnal symptoms not more than twice per month  FEV1 or PEF >80% predicted  PEF or FEV1 variability < 20% |
| --- |
| **Step 2: Mild persistent**  Symptoms more than once per week but less than once a day  Exacerbations may affect activity and sleep  Nocturnal symptoms more than twice per month  FEV1 or PEF >80% predicted  PEF or FEV_1_ variability 20-30% |
| **Step 3: Moderate persistent**  Symptoms daily  Exacerbations may affect activity and sleep  Nocturnal symptoms more than once per week  Daily use of short-acting ß-agonists  FEV_1_ or PEF 60-80% predicted  PEF or FEV_1_ variability >30% |
| **Step 4: Severe persistent**  Symptoms daily  Exacerbations frequent  Frequent nocturnal symptoms  Limitation of physical activities  FEV_1_ or PEF <60% predicted  PEF or FEV_1_ variability >30% |

***^†^*** *Adapted from GINA 2005: Global Strategy for Asthma Management and Prevention - Updated 2005. (*[*http://www.ginasthma.com/Guidelineitem.asp?l1=2&l2=1&intId=1169&archived=1*](http://www.ginasthma.com/Guidelineitem.asp?l1=2&l2=1&intId=1169&archived=1)*, Accession date 16 May 2007)*
