# Supplementary material for: Prescribing patterns of asthma controller therapy for children in UK primary care: a cross-sectional observational study
Source: BMC Pulm Med. 2010 May 14;10:29. doi: 10.1186/1471-2466-10-29 (PMC2882363; doi:10.1186/1471-2466-10-29)
Supplement: Additional file 2 — Criteria for identifying a medical diagnosis of asthma or wheezing. Criteria for identifying a medical diagnosis of asthma or wheezing criteria for identifying children with asthma or wheezing in the GPRD. [file 1471-2466-10-29-S2.DOC]

### Additional file 2 Criteria for identifying a medical diagnosis of asthma or wheezing

| **Criteria** | **Specification** |
| --- | --- |
| Diagnosis of asthma | READ/OXMIS |
| Hospitalisation for asthma | 1 hospitalisations with a primary or secondary diagnosis of asthma (READ/OXMIS) |
| Emergency Department visit for asthma | 1 ED visits with a diagnosis of asthma (READ/OXMIS) |
| Outpatient care visit for asthma | 1 outpatient visits for asthma (READ/OXMIS), including physician visits, laboratory care or ancillary services |
| Pharmacy Rx | 2 claims for a short-acting bronchodilator (either short-acting beta agonist or ipratropium bromide) at least 28 days apart |
| Diagnosis of wheezing | 2 episodes (codes) of wheezing at least 28 days apart |
| Hospitalisation for wheezing | 2 hospitalisations with a primary or secondary diagnosis of wheezing (READ/OXMIS) at least 28 days apart |
| Emergency Department visit for wheezing | 2 ED visits with a diagnosis of wheezing (READ/OXMIS) at least 28 days apart |
| Outpatient care visit for wheezing | 2 outpatient visits for wheezing (READ/OXMIS), including physician visits, laboratory care or ancillary services at least 28 days apart |
