# Supplementary material for: Prescribing patterns of asthma controller therapy for children in UK primary care: a cross-sectional observational study
Source: BMC Pulm Med. 2010 May 14;10:29. doi: 10.1186/1471-2466-10-29 (PMC2882363; doi:10.1186/1471-2466-10-29)
Supplement: Additional file 3 — Table S5 for the manuscript Table S5: Characteristics of 10,004 children new to asthma controller therapy, stratified by treatment group (column percentages). original table S5. [file 1471-2466-10-29-S3.DOC]

**Table 5**: Characteristics of 10,004 children new to asthma controller therapy, stratified by treatment group (column percentages)

| **Characteristics** | **ICS**  **(n = 9059)** | **ICS-LABA**  **(n = 698)** | **LTRA**  **(n = 91)** | **ICS-LTRA**  **(n = 55)** | **Other***  **(n = 101)** | **p value** |
| --- | --- | --- | --- | --- | --- | --- |
| Mean age (SD), yr | 7.9 (3.8) | 10.3 (2.9) | 6.0 (4.1) | 6.1 (3.4) | 9.3 (3.5) | <0.001 |
| Age 0–2 yr | 762 (8.4%) | 8 (1.2%) | 26 (28.6%) | 7 (12.7%) | 3 (3.0%) | <0.001 |
| Age 3–5 yr | 2054 (22.7%) | 43 (6.2%) | 23 (25.3%) | 21 (38.2%) | 14 (13.9%) |
| Age 6–8 yr | 2113 (23.3%) | 141 (20.2%) | 16 (17.6%) | 16 (29.1%) | 21 (20.8%) |
| Age 9–11 yr | 2152 (23.8%) | 204 (29.3%) | 10 (11.0%) | 4 (7.3%) | 32 (31.7%) |
| Age12–14 yr | 1978 (21.8%) | 302 (43.3%) | 16 (17.6%) | 7 (12.7%) | 31 (30.7%) |
| Male sex | 5368 (59.3%) | 414 (59.3%) | 59 (64.8%) | 37 (67.3%) | 64 (63.4%) | 0.514 |
| Median asthma duration (range), yr | 3.5 (0.0–15.0) | 5.9 (0–14) | 2.2 (0.0–12.4) | 2.5 (0.0–11.8) | 4.5 (0.0–12.7) | <0.001 |
| Oral steroid at cohort entry | 511 (5.6%) | 37 (5.3%) | 1 (1.1%) | 4 (7.3%) | 3 (3.0%) | -- |
| Any lung function test at index date | 1457 (16.1%) | 154 (22.1%) | 11 (12.1%) | 8 (14.6%) | 27 (26.7%) | <0.001 |
| Any lung function test up to 6 mo prior | 863 (9.5%) | 91 (13.0%) | 10 (11.0%) | 4 (7.3%) | 6 (5.9%) | 0.043 |
| PEFR, n | 1384 | 153 | 11 | 8 | 25 |  |
| Median PEFR (range), L/min | 243 (2–540) | 260 (80-600) | 240 (80–426) | 230 (110–450) | 254 (70–400) | 0.316 |
| **History of concomitant conditions up to 24 mos before index controller prescription** | | | | | | |
| Dx: sinusitis | 46 (0.5%) | 8 (1.2%) | 0 | 1 (1.8%) | 2 (2.0%) | -- |
| Dx: otitis media | 1382 (15.3%) | 72 (10.3%) | 14 (15.4%) | 7 (12.7%) | 13 (12.9%) | 0.023 |
| Dx: gastro-oesophageal reflux | 53 (0.6%) | 4 (0.6%) | 4 (4.4%) | 0 | 1 (1.0%) | -- |
| Dx: URTI | 4442 (49.0%) | 312 (44.7%) | 54 (59.3%) | 29 (52.7%) | 41 (40.6%) | 0.029 |
| Dx: LRTI | 2325 (25.7%) | 144 (20.6%) | 35 (38.5%) | 21 (38.2%) | 27 (26.7%) | <0.001 |
| Dx/Rx: allergic rhinitis | 907 (10.0%) | 112 (16.1%) | 14 (15.4%) | 5 (9.1%) | 18 (17.8%) | <0.001 |
| Dx/Rx: atopic dermatitis | 3132 (34.6%) | 177 (25.4%) | 36 (39.6%) | 20 (36.4%) | 35 (34.7%) | <0.001 |
| **History of concomitant conditions up to 24 mos before index controller prescription** | | | | | | |
| No. all consultations: 1–4 | 4518 (49.9%) | 358 (51.3%) | 28 (30.8%) | 21 (38.2%) | 42 (41.6%) | <0.001 |
| No. all consultations: 5+ | 3795 (41.9%) | 274 (39.3%) | 60 (65.9%) | 31 (56.4%) | 55 (54.5%) |
| Asthma related visits | 340 (3.8%) | 29 (4.2%) | 10 (11.0%) | 6 (10.9%) | 3 (3.0%) | 0.002 |
| Asthma related visits: 1 | 199 (2.2%) | 17 (2.4%) | 2 (2.2%) | 3 (5.5%) | 1 (1.0%) | -- |
| Asthma related visits: 2+ | 141 (1.6%) | 12 (1.7%) | 8 (8.8%) | 3 (5.5%) | 2 (2.0%) | -- |
| Consultation: LRTI requiring antibiotics | 488 (5.4%) | 20 (2.9%) | 8 (8.8%) | 1 (1.8%) | 8 (7.9%) | -- |
| Oral steroid prescription | 361 (4.0%) | 19 (2.8%) | 7 (7.7%) | 4 (7.3%) | 5 (5.0%) | 0.091 |

Data are n (%) unless otherwise noted.

Dx = diagnosis; ICS = inhaled corticosteroids; LABA = long-acting β2-agonists; LTRA = leukotriene receptor antagonists; LRTI = lower respiratory tract infection; PEFR = peak expiratory flow rate; Rx = recorded prescription; URTI = upper respiratory tract infection. Asthma related visits included hospital and out-of-office visits.

*Other medications included LABA monotherapy, cromones, theophylline, salbutamol-slow release, and therapy combinations for small patient numbers.
